# Non-Displaced Scaphoid Waist Fractures: Percutaneous Screw Fixation Versus Cast Immobilization

**DOI:** 10.7759/cureus.22684

**Published:** 2022-02-28

**Authors:** Serkan Surucu, Lokman Kehribar

**Affiliations:** 1 Orthopaedics/Research Fellow, University of Missouri–Kansas City, Kansas City, USA; 2 Orthopedics and Traumatology, Samsun Gazi State Hospital, Istanbul, TUR

**Keywords:** scaphoid waist fracture, scaphoid fractures, percutaneous osteosynthesis, non-displaced, cast immobilization

## Abstract

Background

Scaphoid waist fractures make up 66% of scaphoid fractures and are mostly non-displaced. The purpose of this study was to demonstrate that percutaneous screw fixation is preferable to cast immobilization in the treatment of non-displaced or minimally displaced scaphoid waist fractures.

Methodology

Between 2017 and 2019, we conducted a retrospective review of patients aged 17-65 years who underwent treatment for acute non-displaced scaphoid waist fractures. In total, 52 patients with scaphoid waist fractures were included in the analysis, 25 of whom underwent percutaneous screw treatment and 27 were treated with a short plaster cast. Patient satisfaction, pain, range of motion, and grip strength were evaluated using the Mayo Modified Wrist Score (MMWS). In addition, the time to return to work/sports, union time, complications, and non-union status were evaluated.

Results

A total of 52 (35 male, 15 female) patients were enrolled in this study. The average follow-up time was 24.9 months (range, 24-29 months). The mean age was 28.12 years (range, 17-45 months). Group 1 consisted of 25 patients who underwent percutaneous screw fixation, and group 2 consisted of 27 patients who were treated with a short plaster cast. There were significant differences in return to work, return to sports, and union time between the two groups (p < 0.001). The sixth-month MMWS was significantly different between the two groups (p < 0.001), but the first-year MMWS was not significantly different between the two groups (p = 0.864). There were no complications in both groups.

Conclusions

With percutaneous screw fixation, acute non-displaced or minimally displaced scaphoid waist fractures demonstrated a high rate of union and early return to work/sports.

## Introduction

Scaphoid fractures are most common in young male athletes between the ages of 15 and 25. The scaphoid is the most often fractured carpal bone, accounting for one-tenth of all hand fractures and two-thirds of all carpal fractures [1,2]. In addition, scaphoid waist fractures account for two-thirds of all scaphoid fractures and are mostly non-displaced [3,4].

Cast stabilization and percutaneous screw osteosynthesis are two treatment options for non-displaced scaphoid waist fractures. Among the reasons for this is that, while the union rate of plaster cast mobilization is comparable to that of percutaneous screw fixation, the plaster cast remains in place for an extended period, delaying a return to sports and other activities [5-7]. The patient group is primarily young and active and wishes to return to sports and social activities as quickly as possible [5].

Except for the systematic review, there has been no study comparing these two treatment methods in the last 10 years. Moreover, systematic reviews evaluate earlier studies. The purpose of this study was to demonstrate that percutaneous screw fixation is superior to cast immobilization for non-displaced or minimally displaced scaphoid waist fractures.

## Materials and methods

This retrospective study was approved by the Samsun Training and Research Hospital (IRB number: 2021/17/15). Between 2017 and 2019, we retrospectively reviewed patients who were managed for acute non-displaced scaphoid waist fractures between the ages of 17 and 65. Patients having a history of an untreated further wrist injury, who were not assessed within two weeks of the injury, or who had a displaced scaphoid waist fracture of more than 1 mm and were smokers were excluded from the study.

The study comprised 52 patients with scaphoid waist fractures, 25 of whom were treated with a percutaneous screw, and 27 were treated with a short plaster cast, according to the files evaluated. Scaphoid fractures in group 1 underwent percutaneous fixation using a volar approach, whereas those in group 2 were treated with a short arm-thumb plaster cast. Following surgery, an approximately two-week-long volar thumb splint was applied.

Patient satisfaction, pain, range of motion, and grip strength were evaluated using the Mayo Modified Wrist Score (MMWS). In addition, the time to return to work/sports, union time, complications, and non-union status were evaluated. For one year, patients were followed consistently. All patients were evaluated one week after treatment started, followed by every two weeks until the fracture healed. To ensure union, all radiographs were re-examined. Following documentation of union, patients were re-evaluated. All patients were examined with MMWS during the sixth-month and first-year follow-up visits.

The data were analyzed using the SPSS version 25 (IBM Corp., Armonk, NY, USA) software package. Descriptive statistics for numerical variables were reported as mean, standard deviation, and median (minimum-maximum), and the number of observations and (percentage) for nominal variables. The Shapiro-Wilk test was used to determine if the distribution of numerical variables was normal or abnormal. The independent samples t-test was used to determine whether there was a statistically significant difference between the two groups regarding normally distributed numerical variables. The Mann-Whitney U test was used to determine whether a statistically significant difference existed in non-normally distributed numerical variables. Nominal variables were examined using the chi-square and Fisher’s exact tests. The results were considered statistically significant at p-values of <0.05.

## Results

A total of 50 (35 male, 15 female) patients were enrolled in this study. The average follow-up time was 24.9 months (range, 24-29 months). The mean age was 28.12 years (range, 17-45 years), and the mean body mass index was 25.32 kg/m^2^ (range, 20-31 kg/m^2^). Group 1 consisted of 25 patients who underwent percutaneous screw fixation, and group 2 consisted of 27 patients who were treated with a short plaster cast. In both groups, the mean age, gender, injured side, hand dominance, follow-up time, and occupation were comparable. In total, 17 scaphoid fractures occurred on the dominant side in group 1, whereas 19 occurred on the dominant side in group 2 (Table 1).

**Table 1:** Demographic characteristics of patients. BMI: body mass index; M: male; F: female; L: left; R: right

	Group 1 (n = 25)	Group 2 (n = 27)	P-value
Age	27.72 ± 7.73	28.64 ± 7.84	0.678
BMI	25.8 (22–31)	24.84 (20–29)	0.300
Gender (M/F)	18 (72%)/7 (28%)	17 (63%)/10 (37%)	0.996
Side of injury (L/R)	10/15	11/16	0.876
Dominant side injury	17	19	0.868
Follow-up time (month)	25.6 (24–27)	27.2 (24–29)	0.886

According to the return to work, return to sports, and union time, there were significant differences between the two groups (p < 0.001). There were significant differences in the sixth-month MMWS between the two groups (p < 0.001), but no significant differences in the first-year MMWS between the two groups (p = 0.864) (Table 2).

**Table 2:** A comparison of the clinical characteristics of the two groups. MMWS: Mayo Modified Wrist Score

	Group 1 (n = 25)	Group 2 (n = 27)	P-value
Return to work (week)	6 (5–8)	9 (8–12)	<0.001
Return to sports (week)	7 (6–9)	11 (9–13)	<0.001
Union time (week)	8 (7–10)	11 (9–13)	<0.001
Non-union	0 (0%)	1 (0.37%)	0.826
Sixth-month MMWS	85.88 ± 4.34	79.88 ± 1.3	<0.001
First-year MMWS	91.68 ± 1.99	89.72 ± 2.28	0.864

## Discussion

Currently, there is a lack of scientific consensus regarding the surgical management of acute non-displaced scaphoid waist fractures [8]. With the advancement of minimally invasive, percutaneous procedures, there has been a shift toward operative management of non-displaced or minimally displaced waist fractures [3]. In this study, we compared the two treatment methods currently in practice for non-displaced scaphoid waist fractures in terms of one-year functional and radiographic outcomes, complications, and non-union. Our findings indicated that, when compared to cast immobilization, surgical treatment resulted in a significantly higher functional score at six months following fracture fixation, but comparable functional outcomes at the first-year follow-up. This was attributable to the fact that screw fixation provides adequate stability and enables increased strength through early wrist mobilization, whereas long-term plaster cast immobilization leads to joint stiffness and muscle weakening [9,10].

According to the literature, 90% of non-displaced or minimally displaced scaphoid waist fractures union within six weeks of adequate cast treatment [3,11]. This treatment can be continued for up to 10 weeks in cases of low compliance [3]. Arora et al. and Bond et al. reported that the mean time to fracture union with percutaneous screw fixation was six to seven weeks, but with cast immobilization this time increased to eleven to twelve weeks. Dinkar et al. reported that all fracture cases treated by percutaneous fixation achieved union within 8.75 weeks (range, 6-12 weeks) [12]. In this study, we found the mean time to union with percutaneous screw fixation was eight weeks (range, 7-10 weeks) and with cast immobilization was 11 weeks (range, 9-13 weeks).

Naranje et al. [13] reported a 100% union rate with percutaneous Herbert screw fixation in 32 patients; we observed a similar result in this study with percutaneous screw fixation. Arora et al. [14] reported one patient with non-union in the operative treatment group, while Bond et al. [15] reported no non-union in both groups. However, it was reported that there was no significant difference between the two groups in these two studies, which is consistent with the meta-analysis by Shen et al. [16]. We observed one non-union with cast immobilization in this study, but there was no statistically significant difference between the two groups, which was consistent with the literature.

McQueen et al. [6] and Saeden et al. [17] reported a significantly shorter time to return to work and sports in patients treated with percutaneous screw fixation in randomized clinical studies comparing conservative and surgical treatment. Clinical assessments were performed at sixth-month and first-year follow-ups using the MMWS in this study. While there was a significant difference between the MMWS in the sixth-month follow-up, the MMWS values of the patients converged at the first-year follow-up. No significant difference between the two groups was reported in studies and the meta-analysis utilizing the Disabilities of the Arm, Shoulder and Hand score [9,18,19].

The limitations of this study were that it was retrospective, had a limited sample size, and did not evaluate cost-effectiveness. Additionally, we did not include individuals’ occupations. A prospective, randomized-controlled study with a larger sample size should be designed including cost analysis.

## Conclusions

At the first-year follow-up, there was no statistically significant difference in patient satisfaction, pain, or MMWS between surgical treatment and cast stabilization for non-displaced or minimally displaced scaphoid waist fractures. However, due to the shorter union time and earlier return to work/sports, percutaneous screw fixation appears to be the favored treatment method.
